# The effects, mechanisms, and influencing factors of concurrent strength and endurance training with different sequences: a semi-systematic review

**DOI:** 10.3389/fspor.2025.1692399

**Published:** 2026-01-23

**Authors:** Zhang Feng, Wang Ying, Wang Jun

**Affiliations:** 1 Shanghai University of Sport, Shanghai, China; 2 Han Dan No. 4 High School, Handan, China; 3 Beijing Sport University, Beijing, China

**Keywords:** concurrent training, molecular biology, neuromuscular adaptation, practical applications, training sequence

## Abstract

Objective: This study investigated how the sequencing of strength and endurance training affects athletic performance, and delved into the underlying mechanisms from the perspectives of neuromuscular and molecular adaptations. Furthermore, factors influencing the effectiveness of concurrent training with different sequences were also analyzed. Design and Methods: A semi-systematic review was conducted following the PRISMA guidelines. Relevant literature was retrieved from PubMed, Web of Science, EBSCO, and CNKI databases using the search terms: “concurrent training”, “simultaneous training”, “combined training”, “concurrent strength and endurance training”, “simultaneous strength and endurance training”, “combined strength and endurance training”, “sequence”, “order”. The retrieval timeframe was from January 1980 to December 2024. Results: Analysis of the 42 included studies revealed that, in human trials, while training sequence generally shows no consistent association with the ultimate gains in endurance, muscle hypertrophy, or maximal strength, adopting a “strength-first” modality optimizes neuromuscular adaptations, thereby enhancing relative strength and explosive power. Although acute molecular responses (e.g., mTOR/AMPK phosphorylation) exhibit sequence-dependent variations, their translation into long-term adaptations is complex and non-linear. Notably, animal experiments demonstrate a far more pronounced regulatory effect of training sequence on hypertrophy-related pathways than human studies, suggesting that species differences and training methodologies may be key contributing factors. Recommendations: The training sequence should be arranged reasonably based on the training objectives and the individual differences among the subjects (e.g., age, training status, sport modality). If the endurance-strength training mode is chosen, it is recommended that the interval between the two types of training be more than 3 h to prevent acute molecular interference. For athletes targeting explosive power or relative strength, the strength-endurance sequence is preferred.

## Introduction

“Concurrent training” refers to the arrangement of strength and endurance training tasks within the same training period or same training session. Its research originated from the report by American exercise physiologist Hickson in 1980 (1). Concurrent strength and endurance training is a widely used method that can effectively improve athletes' endurance and strength in different events (2, 3). However, in concurrent training, residual fatigue and energy substrate consumption after one training session may affect the quality and effectiveness of subsequent training, creating an adverse neuromuscular and molecular environment, thereby impairing the body's adaptive response to training (4, 5). This phenomenon is formally defined as the “interference effect”, which refers to the mutual inhibition of adaptive responses between strength and endurance training when performed concurrently, leading to suboptimal gains in one or both fitness components compared to single-mode training (6). Many factors contribute to the “interference effect”, such as the interval between strength and endurance training, the training level of the subjects, and differences in training intensity and volume, among others. In addition, the sequence of strength and endurance training may be an important factor affecting the outcomes of concurrent training (7). The “sequence effect” is defined as the phenomenon where different sequencing of strength and endurance training (i.e., strength followed by endurance vs. endurance followed by strength) within the same training session or with a short interval between sessions results in distinct training adaptations and performance outcomes (8, 9).

A conceptual framework for the sequence effect within traditional interference theory is as follows: Traditional interference theory focuses on the competitive relationship between strength and endurance training at the physiological and molecular levels (e.g., conflicting energy metabolism pathways, mutually exclusive molecular signaling). The sequence effect extends this theory by emphasizing that the order of training modulates this competitive relationship—specifically, the first training mode occupies key physiological resources (e.g., energy substrates, molecular signaling molecules) and induces initial fatigue, thereby affecting the adaptive response to the subsequent training mode. Understanding the effect of concurrent training sequence on strength and endurance improvement and its potential biological mechanisms can better guide sports training and mass physical activity, providing scientific training methods for enhancing athletes' competitive ability and promoting public health.

## Methods

### Search strategy

Relevant literature was retrieved from four databases: PubMed, Web of Science, EBSCO, and CNKI. The search terms were combined as follows: “concurrent training”, “simultaneous training”, “combined training”, “concurrent strength and endurance training”, “simultaneous strength and endurance training”, “combined strength and endurance training”, “sequence”, “order”. The retrieval timeframe was limited to January 1980 to December 2024, Detailed search strategies are provided in Additional File 1. (A number of non-core sources were included as supplementary references in 2025).

### Inclusion and exclusion criteria

*Inclusion criteria:* (1) Peer-reviewed original research articles, systematic reviews, or meta-analyses; (2) Studies involving human or animal subjects (with clear grouping by training sequence); (3) Studies that clearly describe training protocols (intensity, volume, duration, interval between training modes); (4) Studies reporting relevant outcome indicators (maximal strength, explosive power, VO₂max; molecular indicators: mTOR, AMPK, MuRF-1, electromyographic activity, H-reflex, etc.).

**Figure 1:**
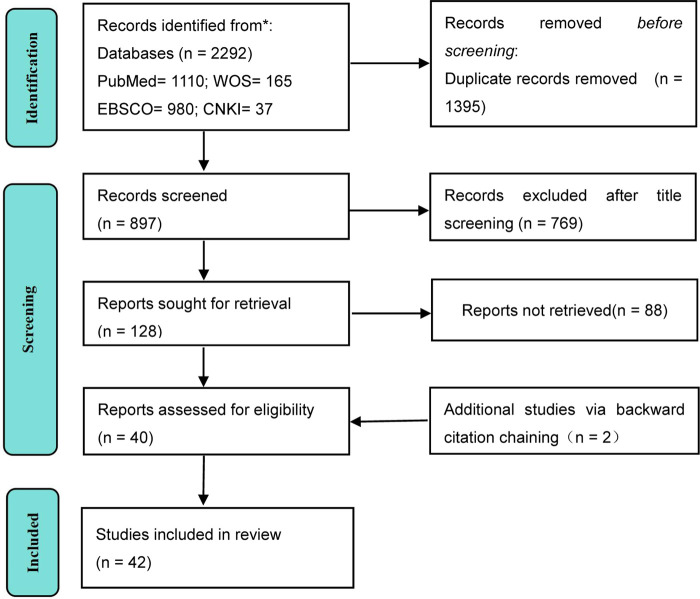
PRISMA flow diagram of the study selection process.

*Exclusion criteria:* (1) Non-peer-reviewed literature (e.g., conference abstracts, dissertations); (2) Studies focusing solely on single-mode training (pure strength or pure endurance training); (3) Studies with incomplete data, unclear experimental design, or unextractable outcome indicators; (4) Duplicate publications; (5) The published language is neither Chinese nor English. (refer to Figure 1).

### Literature screening and quality assessment

Literature screening was performed independently by two researchers (ZF and WY) in three stages: (1) Initial screening: Excluding irrelevant literature based on titles and abstracts; (2) Full-text screening: Reading full texts to confirm compliance with inclusion criteria; (3) Quality assessment: Study quality was assessed independently by two researchers (ZF and WY) using the Newcastle-Ottawa Scale (NOS) for cohort/case-control studies and the AMSTAR 2 scale for systematic reviews/meta-analyses, with disputes resolved by a third researcher (WJ). Detailed quality scores for each included study are presented in Additional File 2.

### Data synthesis

This review was designed as a semi-systematic review, combining systematic literature retrieval and qualitative synthesis. Data extraction included study characteristics (subjects, sample size, training protocol), outcome indicators, and key findings. For consistent findings across multiple studies, a narrative synthesis was conducted; for conflicting findings, the reasons for heterogeneity (e.g., subject characteristics, training parameters) were analyzed. Human and animal studies were synthesized separately to avoid inappropriate generalization, and the differences between the two types of studies were discussed explicitly. Detailed information of included studies are provided in Additional File 2.

## Results

### Quality assessment of included studies and implications for evidence

#### Quality assessment of included studies

A total of 42 studies were included in this review, consistent with the classification in Additional File 2: 29 original research articles (26 human trials, 3 animal studies) and 13 reviews (7 narrative reviews, 6 systematic reviews & meta-analyses).

For the 26 human trials, the Newcastle-Ottawa Scale (NOS, maximum score = 9) was used for assessment. Scores ranged from 6 to 9 (median = 7.5), with 57.69% (15 studies) rated as “high quality” (score ≥8)—characterized by rigorous exposure assessment, accurate outcome measurement, and effective control of confounding factors (e.g., age matching, adjustment for training status, or correction of baseline differences). 38.46% (10 studies) were “moderate to high quality” (score = 6–7), mainly limited by insufficient reporting of outcome assessor blinding or incomplete description of follow-up loss; 3.84% (1 studies) were “low quality” (score = 6), due to unclear definitions of training protocols (e.g., unreported exercise intensity) or small sample sizes (<20 participants per group).

For the 3 animal studies, the Modified Newcastle-Ottawa Scale (adapted for preclinical research, maximum score = 9) was applied. Scores ranged from 7 to 8 (median = 7.6), and all studies were rated “moderate to high quality,” with consistent reporting of grouping methods, intervention details, and outcome indicators. Limitations included 1 study lacking randomization and 2 studies having no description of blinding (consistent with the detailed records in Additional File 2).

Among the 13 reviews, the 7 narrative reviews were not applicable for quantitative quality scoring (marked as “Descriptive assessment only” in Additional File 2), as they do not meet the criteria for tools like NOS or AMSTAR 2. For the 6 systematic reviews & meta-analyses, AMSTAR 2 (focused on critical/major flaws) was used: 33.3% (2 studies) were “high quality” (no critical flaws, with ≥7 non-critical flaws addressed, e.g., comprehensive literature searches, clear inclusion/exclusion criteria, and thorough assessment of publication bias); 50% (4 studies) were “moderate quality” (1–2 critical flaws, e.g., incomplete reporting of study selection processes or unassessed sources of heterogeneity).

#### Implications for evidence interpretation

Overall, 78.6% of included studies (33/42) were of moderate to high quality, which supports the reliability of core findings (e.g., sequence effects on neuromuscular adaptations and explosive power) in this review. Key considerations are as follows:

*Low-quality study handling:* The 1 low-quality human trials (small samples, unclear protocols) were excluded from the narrative synthesis of key outcomes (e.g., muscle hypertrophy, maximal strength) to avoid result bias.
*Animal study extrapolation caution:* Despite their moderate-to-high quality, animal studies have inherent translational limitations (e.g., electrical stimulation in experiments not replicating human strength training). Thus, their molecular pathway findings (e.g., mTOR signaling) should be interpreted cautiously when extended to human populations.
*Data traceability:* Detailed quality assessment data for each study—including study ID, design, assessment tool, total score, item-specific performance, and risk of bias rating—are fully documented in Additional File 2 for reference.

### The effects of concurrent training with different sequences on sports performance

#### Effects on strength performance

Sports training is the most effective way to improve skeletal muscle mass and strength. Different forms of exercise lead to distinct adaptations in skeletal muscle. Therefore, arranging strength and endurance training in a single session may interfere with both qualities, especially with strength quality (5). The order of strength and endurance training may be an important factor affecting skeletal muscle hypertrophy and strength improvement.

#### Effects on skeletal muscle hypertrophy and maximum strength

Skeletal muscle hypertrophy results from skeletal muscle protein synthesis exceeding degradation. Resistance training can increase the number of satellite cells and, simultaneously, lead to a proportional increase in muscle nuclei, thereby promoting muscle fibre hypertrophy (10). Skeletal muscle hypertrophy can effectively improve maximum strength to some extent.

The sequence of strength and endurance in concurrent training is not the leading cause of interference effects on skeletal muscle hypertrophy and maximum strength in human studies (11–13). A systematic review and meta-analysis by Schumann et al. (14) (*n* = 43 studies) showed that regardless of the type of aerobic training (cycling vs. running), the frequency of concurrent training (>5 vs. <5 times a week), training status (trained vs. untrained), average age (<40 vs. >40 years old), and training mode (same training session vs. same day vs. different days), concurrent training with different sequences did not interfere with skeletal muscle hypertrophy and maximum strength. However, this study has limitations: most included studies had small sample sizes (<30 subjects per group), and the training duration was relatively short (8–12 weeks), which may limit the detection of potential sequence effects. Previously, Schumann et al. (8) found in a study on young untrained men (*n* = 42) that concurrent training of strength and endurance in both sequences had similar effects on serum-related hormone levels (testosterone, cortisol, growth hormone), indicating no correlation between training sequence and skeletal muscle protein synthesis. This conclusion was re-verified by Lundberg et al. (15) through another systematic review (*n* = 15 studies), but the authors noted that the heterogeneity of training volume and intensity across studies may mask potential sequence effects.

In studies on older adults, Cadore et al. (16, 17) (both *n* = 26) conducted 12-week concurrent training programs and found that both sequences effectively increased the thickness of upper and lower limb skeletal muscles, with no significant difference between groups (*p* > 0.05). Wilhelm et al. (18) (*n* = 36 older men) implemented a 12-week concurrent training program (twice weekly) and assessed knee extension 1RM and muscle ultrasound measurements. All indicators improved significantly compared to baseline (*p* ≤ 0.05), but no significant differences were observed between sequence groups (*p* ≥ 0.05). However, these studies had relatively small sample sizes and lacked long-term follow-up, so the conclusions may not be generalizable to larger populations. Chtara et al. (19) (*n* = 48 physical education majors) found that although pure strength training contributed more to strength improvement than concurrent training, the sequence of concurrent training was not the main factor affecting maximum strength. Makhlouf et al. (20) (*n* = 57 young elite-level male field soccer players) reported that same-day concurrent training had similar or better effects on muscle strength compared to alternate-day training, and the sequence was irrelevant to the training effect. It is recommended that coaches arrange strength and endurance training in daily training according to needs, without considering the sequence.

In contrast, animal studies have shown different results. Shirai et al. (21) used electrical stimulation to simulate strength training and treadmill running for endurance training, and found that the strength-endurance sequence had a more noticeable impact on the skeletal muscle hypertrophy signal (mTOR) and was more conducive to skeletal muscle hypertrophy (22). However, the limitation of animal studies is that electrical stimulation cannot fully replicate human strength training (e.g., squats, deadlifts), and the metabolic and physiological responses of rats may differ from humans, leading to inconsistent conclusions between animal and human studies.

#### Effects on explosive power

The strength-endurance sequence is optimal for explosive power development. This view holds that in arranging concurrent training, the fatigue generated by the first part will affect the quality of the latter part, leading to a decline in performance. Some scholars refer to this as the fatigue accumulation effect (5). According to this view, the arrangement of the concurrent training sequence should be related to the training purpose, with the quality that needs to be improved first arranged in the first half of the concurrent training to avoid the fatigue accumulation effect. Craig et al. (23) (*n* = 36 young men) found in a study on the effect of concurrent training sequence on strength development that performing strength training immediately after endurance training reduces the quality, load, and intensity of strength training due to residual fatigue. It was also pointed out that the fatigue generated by previous endurance training might weaken the ability of trained muscles to generate sufficient muscle tension during strength training, as shown in a review by Schumann et al. (14) believed that although skeletal muscle fatigue caused by long-term endurance training does not affect maximum strength, it significantly reduces the improvement of explosive power. The sequence of strength training first and then endurance is more conducive to improving subjects' countermovement jump, explosive power, and muscle work ability (12). Alves et al. (24) (*n* = 128 prepubescent boys) used prepubescent male subjects in the study. They found that the strength-endurance training sequence is more conducive to improving the subjects' explosive power (medicine ball throw, countermovement jump, standing long jump). In addition, two studies by Cadore et al. (16, 17) both suggested that the sequence of strength followed by endurance is more conducive to adapting the nervous system in older adults, thereby promoting the improvement of explosive power.

#### Effects on aerobic and anaerobic endurance performance

Current evidence suggests that concurrent training does not interfere with aerobic endurance. Hickson (1) showed in the first research conclusion on concurrent training that the maximum oxygen uptake of subjects in the endurance training group and the concurrent training group increased by 17% and 20%, respectively, indicating that concurrent training has no negative impact on aerobic endurance, which has also been recognised by other scholars (25, 26). The academic community has reached a relatively consistent conclusion regarding whether the sequence of concurrent training leads to differences in the improvement of aerobic endurance. The improvement of aerobic endurance levels is unrelated to the sequence of strength and endurance training (5, 13, 27). Eddens and his colleagues (28) found that the endurance-strength and strength-endurance groups significantly improved indicators such as maximum oxygen uptake and body fat percentage after exercise intervention. However, no significant difference was found between the two experimental groups. At the same time, Vilaça-alves et al. (29) showed that after subjects received concurrent training interventions with different sequences, their maximum oxygen uptake and other indicators were tested. It was found that both indicators were significantly improved after the training intervention, with no difference between the sequences. Wilhelm et al. (18) believed that both sequences of concurrent training could improve maximum aerobic capacity (maximum oxygen uptake and the second ventilatory threshold, VT2). However, there was no significant difference between the different sequence groups. The reason may be that the improvement in endurance quality is closely related to the training volume. No matter how the sequence changes, the total amount of training intervention remains unchanged, so the stimulation of the body's aerobic metabolism capacity remains similar. In addition, compared to pure endurance training, concurrent training will not harm maximum oxygen uptake (11) but will increase the proportion of type IIa muscle fibres (30). At the same time, strength training can increase skeletal muscle stiffness, improve neuromuscular function, and enhance the coordination and control abilities of the neuromuscular system. Improving these functions can enhance athletes' running economy (31), thus promoting the effective improvement of subjects' aerobic capacity.

Few studies have explored the impact of concurrent training sequence on anaerobic metabolism, but anaerobic metabolism is crucial for strength performance and explosive power. Recent studies have shown that the strength-endurance sequence may be more conducive to improving anaerobic metabolism indicators. For example, Ramirez-campillo et al. (32) (*n* = 36 young soccer players) found that the strength-plyometric sequence significantly improved anaerobic power (measured by the Wingate test) compared to the plyometric-strength sequence. This may be because strength training first activates fast-twitch muscle fibres and enhances anaerobic energy supply capacity, while prior endurance training induces fatigue and impairs anaerobic performance during subsequent strength training. However, more studies are needed to confirm this conclusion, especially in different sports and population groups.

### Biological mechanisms of concurrent training with different sequences in improving strength and endurance qualities

#### Neuromuscular adaptations to concurrent training with different sequences

Good adaptation of the neuromuscular system to training can improve corresponding physical qualities, and different training methods lead to distinct neuromuscular adaptations. Strength training-induced central nervous system adaptations are mainly manifested in: increased output frequency of central nervous impulses, enhanced connection of spinal interneurons, improved ability of motor neurons to switch between excitation and inhibition (33), and greater corticospinal drive (34). These adaptations enhance the coordination and control of motor units, improving strength output efficiency. After endurance training, subjects' H-reflex increases, anti-fatigue ability is enhanced, and the activity of *α* motor neurons increases, mobilizing more slow-twitch muscle fibres to participate in exercise and delaying fatigue (35, 36).

The sequence of concurrent training may affect the improvement of strength, but has no impact on the increase in muscle mass (28), indicating that, regardless of the training sequence, endurance training does not affect the skeletal muscle hypertrophy caused by strength training. From this, we infer that the impact of concurrent training with different sequences on strength quality may be related to the nervous system's adaptation. Studies have shown that sports performance seems to have little to do with the sequence of strength and endurance, but there are differences in the observation of neural adaptation between the two groups (37) (*n* = 56 middle-aged adults). When older adults were the research subjects, it was found that in concurrent training three times a week, when strength training was performed before endurance training, the neuromuscular system produced better adaptation (38). This conclusion was also verified in an experiment involving middle-aged subjects (37). These studies suggest that arranging strength training before endurance training will lead to good adaptation in the nervous system. However, the opposite sequence may disrupt the nervous system. If aerobic training is always scheduled before strength training, it may not impact the improvement of maximum strength. However, at least to some extent, it will have a specific inhibitory effect on the nervous system (39) (*n* = 34 young adults). Relative muscle strength can effectively reflect the nervous system's role in the process of strength development, and an increase in relative muscle strength indicates good adaptation of the nervous system to training (40–42). Cadore et al. (17) (*n* = 30 older men) studied and concluded that, compared to the opposite training sequence, the program with strength training first resulted in a greater increase in relative muscle strength (27% vs. 15%, respectively).

To sum up, the interference effect can be partially explained by the fact that neural adaptation is affected after strength training. From a practical point of view, although concurrent training may interfere with neuromuscular adaptation, arranging strength training at the beginning of a session reduces this adverse impact. That is, it can maximise neuromuscular adaptation during concurrent training.

### Molecular biological adaptation to concurrent training with different sequences

Different training forms induce distinct molecular biological responses. Resistance training-induced skeletal muscle hypertrophy is related to the mTOR-centered molecular pathway; endurance training improves aerobic endurance mainly through molecules such as AMPK, CaMK, and p38MAP to activate PGC-1*α*, leading to changes in mitochondrial quantity and function.

#### Effects on energy metabolism and AMPK activation

**Figure 2:**
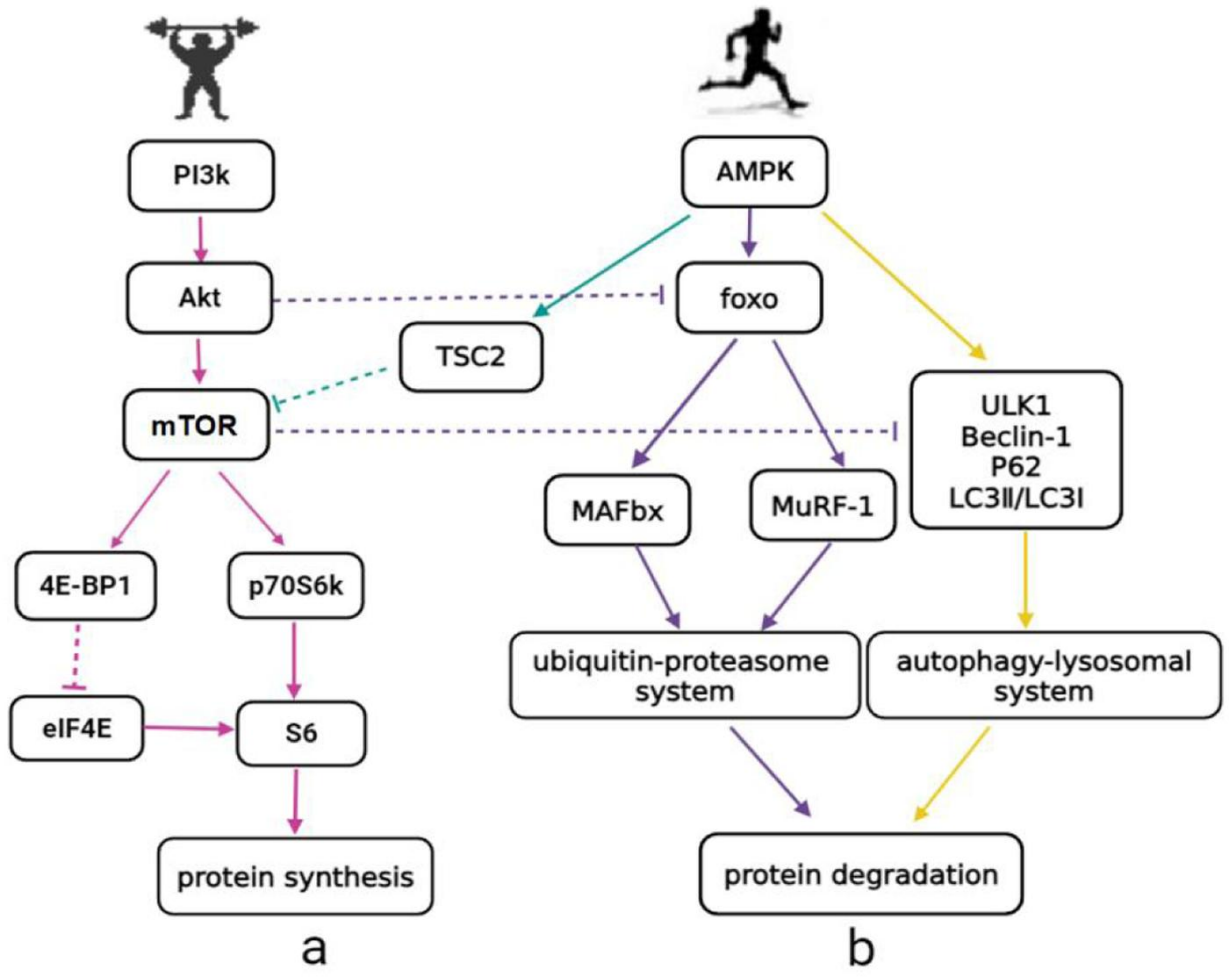
Molecular pathway of skeletal muscle protein metabolism induced by strength and endurance training [adapted from Coffey (56), Hawley (57)]. **(a)** Green line: AMPK/TSC2/mTORC1 pathway, which inhibits protein synthesis; PI3K, phosphatidylinositol 3-kinase; Akt, protein kinase b; mTOR, mammalian target of rapamycin; 4E-BP1, eukaryotic translation initiation factor 4E binding protein 1; p70S6K, p70 ribosomal S6 kinase; eIF4E, eukaryotic translation initiation factor 4E; S6, ribosomal protein S6; TSC2, tuberous sclerosis complex 2. **(b)** Purple line: AMPK/FOXO/MURF-1/MAFbx pathway, which promotes protein degradation. AMPK, AMP-activated protein kinase; foxo, forkhead box O; MAFbx, muscle atrophy F-box; MuRF-1, muscle RING-finger protein 1; ULK1, unc-51 like autophagy activating kinase 1; Beclin-1, beclin 1 autophagy related; P62, sequestosome 1; LC3II/LC3I, microtubule-associated protein 1 light chain 3 II/I.

Endurance training leads to significant changes in energy metabolism: increased ATP turnover results in a rise in AMP levels (e.g., via the adenylate kinase reaction: 2ADP ↔ ATP + AMP), altering the ATP/AMP ratio and stimulating AMPK phosphorylation (43). AMPK acts as an energy sensor and affects skeletal muscle protein metabolism through three pathways: (1) Inhibiting mTOR phosphorylation through TSC2, reducing protein synthesis rate (Figure 2a); (2) Increasing protein degradation through the ubiquitin-proteasome system; (3) Promoting protein degradation through the autophagy-lysosome system (Figure 2b). This indicates that strength and endurance training may conflict at the molecular level, interfering with skeletal muscle hypertrophy or strength (26, 44).

#### Effects on the skeletal muscle protein synthesis pathway

The Akt/mTOR/p70S6K/S6 pathway is the primary molecular mechanism for exercise-induced skeletal muscle protein synthesis. Ogasawara et al. (45) (rats) found that the endurance-strength sequence resulted in higher phosphorylation levels of mTOR, p70S6K, and S6 compared to the strength-endurance sequence, suggesting that the last training form determines the molecular response. However, this conclusion was derived from animal studies, and human studies have shown inconsistent results. Coffey et al. (46) (*n* = 8 young men) found that the phosphorylation level of S6 was higher after strength training than endurance training in the first module of concurrent training, and the endurance-strength group had higher S6 phosphorylation 3 h post-exercise. However, the sample size of this study was small, and the results may not be generalizable. Studies on Akt molecules have shown that concurrent training with different sequences increases Akt phosphorylation, which is unrelated to sequence (22), and the activation of Akt and its downstream molecules is not synchronized, possibly due to non-Akt-dependent protein synthesis pathways (47, 48).

Another protein synthesis pathway is mTORC1/4E-BP1/eIF4E/S6 (Figure 2a). 4E-BP1 is a translation-inhibitory protein that binds to the 5′ cap structure of mRNA, inhibiting translation initiation. High phosphorylation of 4E-BP1 releases the cap structure, promoting protein synthesis (49). Shirai et al. (21, 22) (rats, *n* = 40 per study) found that the RE-MICT sequence group had higher 4E-BP1 phosphorylation than the control group and the opposite sequence group, but no difference was observed when HIIT was used instead of MICT. This indicates that training method is also a factor affecting the interference effect. No significant changes in Akt, mTOR, and TSC2 were found between groups, suggesting that 4E-BP1 may be regulated by other molecular pathways.

#### Effects on the skeletal muscle protein degradation pathway

The AMPK/TSC2/mTORC1 pathway (green line in Figure 2a) reduces protein synthesis rate. Aerobic exercise activates AMPK in skeletal muscle cells (50), and AMPK inhibits mTORC1 activity through TSC2 or direct phosphorylation, blocking downstream signaling and hindering protein synthesis (51). AMPK is highly sensitive to exercise intensity: its activity increases at the start of exercise and returns to baseline shortly after exercise (52, 53). This inhibitory effect decreases or disappears if the interval between training modes is too long, so the interval is crucial for controlling skeletal muscle protein synthesis.

The strength-endurance sequence enhances TSC2 phosphorylation more than the reverse sequence, consistent with mTOR activity trends (46), supporting the conclusion that mTOR regulation depends on TSC2 signaling. Coffey et al. (54) (*n* = 6 young men) found that mTOR Ser2448 phosphorylation was significantly suppressed when strength training preceded endurance training, but not in the opposite sequence, which may be due to AMPK activation during endurance training inhibiting mTOR phosphorylation.

The AMPK/FOXO/MURF-1/MAFbx pathway (purple line in Figure 2b) accelerates protein degradation. The ubiquitin-proteasome system plays an important role in protein metabolism, and MURF-1 and MAFbx are muscle-specific E3 ubiquitin ligases (55). Coffey et al. (54) found that MAFbx did not change significantly after concurrent training, and MuRF-1 gene expression increased 3 h post-exercise, unrelated to sequence. In another study, Coffey et al. (46) found that MuRF-1 increased significantly 3 h post-exercise in the strength-endurance group compared to the endurance-strength group, indicating that the strength-endurance sequence may increase protein degradation rate, which is not conducive to skeletal muscle hypertrophy. The inconsistent conclusions between the two studies may be due to individual differences in subjects and different sampling times.

It should be noted that most molecular mechanism studies use animal subjects and focus on single or partial molecular pathways, leading to potential one-sided conclusions. There are significant differences between humans and animals in training interventions and physiological responses, which may explain the inconsistency between human performance and animal molecular expression. In addition, the role of autophagy in skeletal muscle protein degradation cannot be ignored, but relevant literature on concurrent training is limited, requiring further research.

#### Effects on indicators related to aerobic metabolism

Aerobic metabolism indicators are related to aerobic exercise capacity. Chtara et al. (19) (*n* = 48 physical education majors) found that after 12 weeks of concurrent training, the levels of citrate synthase (CS) and cytochrome C oxidase in skeletal muscle increased significantly, and these changes were related to training sequence. However, Shirai et al. (22) (rats) found that both sequences increased liver glycogen content, with no significant difference between groups. Mitochondrial biogenesis markers (e.g., PGC-1*α*, SDHB, UQCRC) were significantly improved in both sequence groups compared to the control group, but no difference was observed between sequences (21, 22). These results further confirm that endurance improvement is unrelated to training sequence.

### Influencing factors of concurrent training effects with different sequences

Concurrent strength and endurance training is a complex training method whose effectiveness is influenced by multiple factors, such as exercise mode, intensity, training interval, participants' training status (e.g., elite athletes vs. sedentary individuals, young vs. older adults), and individual differences. In practical training, these factors should be fully considered to avoid confounding effects from non-target factors, and training protocols should be adjusted accordingly to maximise effectiveness.

#### Subject differences

Differences in age and training level may lead to inconsistent research conclusions. Two meta-analyses (5, 28) found that concurrent training sequence has different effects on adults and adolescents: adults achieve better dynamic strength improvement with the strength-endurance sequence, while adolescents benefit more from the aerobic-strength sequence. This may be because adolescents have stronger anti-fatigue ability and faster recovery after high-intensity training (58). For older adults, the strength-endurance sequence maximizes neuromuscular adaptations, resulting in better maximum dynamic strength and relative muscle strength (16, 17). Cadore et al. (17) recommended that older adults adopt the strength-first sequence to improve muscle mass and health-related functions.

Training status is another key factor: untrained individuals can achieve significant benefits regardless of sequence (56), possibly due to lower motor function and greater adaptation potential. For elite athletes, the sequence effect may be more pronounced due to their higher training level, but relevant studies are limited.

#### Training purposes

The training sequence should prioritize the primary goal when the body is not fatigued (59). For improving relative strength, explosive power, or muscle mass, the strength-endurance sequence is recommended (36, 60). Yu Hongjun (61) pointed out that the sequence effect is related to sport type: the endurance-strength sequence is suitable for running events, while the strength-endurance sequence is better for rowing and canoeing. For improving aerobic endurance, the sequence is less critical, but attention should be paid to the training interval.

#### Exercise modes

Different strength and endurance training modes affect the sequence effect (21, 22). Arranging strength training after low-intensity endurance training (non-energy-exhausting) can improve endurance and maximum strength without interfering with molecular signaling (62–64). Ramirez-campillo et al. (32) (*n* = 42 young soccer players) found that the sequence of plyometric training and soccer-specific training had no significant effect on strength improvement. HIIT combined with strength training may yield greater benefits, especially for amateur subjects (65).

The training environment also affects outcomes. Casuso et al. (66) (*n* = 18 athletes) found that sprint interval swimming induced less inflammatory response than running. This suggests that swimming as endurance training reduces body load compared to running, resulting in less interference with subsequent strength training. Wilson et al. (67) (meta-analysis, *n* = 21 studies) found that running-based endurance training had a more obvious interference effect on muscle hypertrophy than cycling, possibly due to eccentric contraction-induced muscle damage in running.

#### Interval time between strength and endurance

The interval between training modes is crucial for molecular signaling (63). After high-intensity endurance training, AMPK increases rapidly and takes at least 3 h to return to baseline (68). mTORC1 activity lasts 18 h after strength training (69). Therefore, the endurance-strength sequence requires an interval of at least 3 h to eliminate molecular interference. In addition, fatigue recovery should be considered: Doma et al. (70) (*n* = 12 resistance-untrained men) found that running economy decreased within 8 h after lower limb strength training, so the interval should be adjusted based on training intensity.

## Discussion

### Main findings

This semi-systematic review found that the concurrent training sequence has no significant effect on aerobic endurance, skeletal muscle hypertrophy, or maximum strength in most human trials, but the strength-endurance sequence is more conducive to neuromuscular adaptations, improving relative strength and explosive power. Molecular responses (e.g., mTOR, AMPK phosphorylation) show sequence dependence, but the translation from acute signals to long-term adaptations is non-linear. Animal studies demonstrate more pronounced sequence effects on molecular pathways than human studies, which may be due to differences in training interventions and species-specific responses.

### Comparison with previous studies

Our findings are consistent with Schumann et al. (14) and Eddens et al. (28), confirming that the sequence has no significant effect on muscle hypertrophy and maximum strength. However, we emphasize the difference between molecular and performance outcomes, which was not fully discussed in previous reviews.

### Limitations

This review has several limitations: (1) Heterogeneity of training modes: The included studies used various training protocols (intensity, volume, duration), which may affect the sequence effect; (2) Inconsistency between human and animal studies: Animal studies cannot fully replicate human training, leading to potential differences in conclusions; (3) Limited sample sizes: Most studies had small sample sizes (<30 subjects per group), reducing statistical power; (4) Short intervention durations: Most studies lasted 8–12 weeks, and long-term sequence effects remain unclear; (5) Lack of mechanistic studies in humans: Most molecular mechanism studies used animal subjects, limiting the generalization to humans; (6) Few studies on elite athletes: The conclusions are mainly based on healthy young or older adults, with limited applicability to elite athletes.

## Conclusions and recommendations

Current evidence suggests that the improvement of aerobic endurance, skeletal muscle hypertrophy, and maximum strength is unrelated to the concurrent training sequence in most human trials. However, the strength-endurance sequence enhances neuromuscular adaptations, benefiting relative strength and explosive power. Molecular responses show sequence dependence, but acute molecular signals do not linearly translate to long-term chronic adaptations. Animal studies have more pronounced sequence effects on molecular pathways than human studies, which should be interpreted cautiously.

*Practical recommendations:* Based on training objectives: (1) For improving relative strength, explosive power: Adopt the strength-endurance sequence, with strength training arranged in the non-fatigued state; (2) For improving aerobic endurance: The sequence is less critical, but ensure sufficient total training volume.

Based on individual differences: (1) Older adults and adults: Strength-endurance sequence is recommended to maximize neuromuscular adaptations; (2) Adolescents: Aerobic-strength sequence may be more beneficial; (3) Untrained individuals: Sequence is less important, focus on gradually increasing training volume; (4) Elite athletes: Conduct individualized training based on sport type and training status.

Training interval and mode: (1) Endurance-strength sequence: Ensure an interval of at least 3 h to eliminate molecular interference; (2) Low-intensity endurance training can be arranged before strength training without obvious interference; (3) Choose appropriate endurance training modes: Cycling or swimming may reduce interference compared to running; (4) Combine HIIT with strength training for better comprehensive benefits.

Future research directions: (1) Conduct large-sample, long-term follow-up studies to explore the long-term sequence effect; (2) Strengthen human mechanistic studies to clarify the molecular mechanism of the sequence effect; (3) Focus on elite athletes and special populations to improve the applicability of conclusions; (4) Explore the interaction between training sequence and other factors to provide more comprehensive training guidance.
