# Supplementary material for: The effects, mechanisms, and influencing factors of concurrent strength and endurance training with different sequences: a semi-systematic review
Source: Front Sports Act Living. 2026 Jan 23;7:1692399. doi: 10.3389/fspor.2025.1692399 (PMC12885173; doi:10.3389/fspor.2025.1692399)
Supplement: Supplementary file 1 [file Table1.docx]

**Additional file 1: Search strategy**

| Database | Run dates | Search strategy | Search result |
| --- | --- | --- | --- |
| Web of Science | 2024-12 | (concurrent training (Abstract) OR simultaneous training (Abstract) OR combined training (Abstract) OR concurrent strength and endurance training (Abstract) OR simultaneous strength and endurance training (Abstract) OR combined strength and endurance training (Abstract) ) AND (sequence (Abstract) OR order (Abstract)) Retrieval Timeframe: January 1980 - December 2024 | 165 |
| EBSCO | 2024-12 | (concurrent training OR simultaneous training OR combined training OR concurrent strength and endurance training OR simultaneous strength and endurance training OR combined strength and endurance training) AND (sequence OR order) Filter criteria: Full-text, Scholarly Journals; Retrieval Timeframe: January 1980 - December 2024 | 980 |
| PubMed | 2024-12 | ((concurrent training [Title/Abstract] OR simultaneous training [Title/Abstract] OR combined training [Title/Abstract] OR concurrent strength and endurance training [Title/Abstract] OR simultaneous strength and endurance training [Title/Abstract] OR combined strength and endurance training [Title/Abstract]) AND (sequence [Title/Abstract] OR order[Title/Abstract])) Retrieval Timeframe: January 1980 - December 2024 | 1110 |
| CNKI | 2024-12 | （同期训练（篇关摘）OR联合训练（篇关摘）OR同期力量和耐力训练（篇关摘）OR力量与耐力同期训练（篇关摘）OR力量耐力组合训练（篇关摘））AND （训练顺序（篇关摘））OR（运动顺序（篇关摘））, 学科限定为：体育; 时间限定: 1980年1月-2024年12月 | 37 |
